# Cobalt Catalyzed C-P Bond Formation by Cross-Coupling of Boronic Acids with P(O)H Compounds in Presence of Zinc

**DOI:** 10.3390/molecules25020290

**Published:** 2020-01-10

**Authors:** Ian Hicks, Jonathan McTague, Tatiana Hapatsha, Rania Teriak, Parminder Kaur

**Affiliations:** Department of Chemistry, College of Science and Health, William Paterson University, 300 Pompton Road, Wayne, NJ 07470, USA; hicksi1@student.wpunj.edu (I.H.); mctaguej@student.wpunj.edu (J.M.); hapatshat@student.wpunj.edu (T.H.); teriakr@yahoo.com (R.T.)

**Keywords:** cross-coupling reactions, terpyridine, arylboronic acid, alkyl/aryl phosphonates, tridendate ligand

## Abstract

In our current work, we have reported the first cobalt-catalyzed cross-coupling of arylboronic acid with alkyl/aryl phosphites under mild conditions. The reaction was carried out in the presence of zinc powder as an additive and ter-pyridine as a ligand. The use of non-precious cobalt salt makes the protocol advantageous, as it is inexpensive and more abundant than the previously used methods where precious metal salts (Pd and Pt) were used. The reaction has a wide substrate scope and the products were obtained in good yields.

## 1. Introduction

Organophosphorus compounds have great significance due to their extensive use in various fields such as medicine, agriculture, polymer science, and material chemistry [1,2,3,4,5,6,7,8,9,10,11,12,13,14,15,16,17,18,19,20,21,22,23,24,25]. The wide application of organophosphorus compounds varies from their use as catalysts in various organic transformations, to being important additives in lubricants [5,6]. Organophosphorus compounds are also important building blocks for nucleic acid chemistry and are associated with increased biological activity due to the presence of the C-P bond [1,2,3,4,5,6,7,8,9,10,11]. Hence, there is growing interest in developing new methodologies for the synthesis of the C-P bond. In addition to the classical methods documented in the literature [12,13,14,15,16,17,18,19,20,21,22,23,24,25], several new transition-metal-catalyzed approaches have been developed, mainly for the synthesis of triarylphosphine compounds via cross-coupling reactions of trialkylphosphine with different aryl sources in the presence of metals such as Ni-, Cu-, and Pd. [13,14,15] Among the various aryl partners, arylboronic acid is a popular choice for various C-C and C-heteroatom bond formation reactions via different cross-coupling methodologies. However, despite the extensive use of arylboronic acid in a variety of other cross-coupling reactions [26,27,28], it has not been used quite as often in the literature for the C-P bond formation and only a couple of reports have been published so far. In 2008, Stawinski and his group published one of the first palladium-catalyzed cross-coupling reactions of aryl boronic acid with dialkyl phosphites [29]. The method showed a moderate to good yield of the product, but the substrate scope was limited. Moreover, the reaction was done under microwave irradiation in the presence of DMF as solvent and *p*-benzoquinone as oxidant. More recently, Zhao et al. reported a copper-catalyzed Chan–Lam-type reaction of arylboronic acid with H-phosphonate diesters. This reaction was carried out in the presence of 1,10-phenanthroline as the ligand. However, while the reaction resulted in moderate to high yields of the products, it was still limited to certain dialkyl phosphites. Only a poor reaction yield was observed with the dibenzyl phosphite [30]. Most of the reactions were reported with diethyl phosphite, and some of these reactions suffered poor product yields. Additionally, the protocol requires the use of ligand (1,10-phenanthroline) for the catalysis [30]. Later, Zhao et al. reported the nickel-catalyzed cross-coupling of arylboronic acid with secondary phosphine oxide for the synthesis of triarylphosphine oxides [31]. While moderate to high yields of the products were obtained, the reaction was performed under high catalyst loadings, as well as elevated temperature (100 °C). Hence, it is of great interest to find an efficient, inexpensive, and more versatile, non-precious metal-based catalytic system for the synthesis of valuable C-P bond formation. Among other metals, cobalt has been widely used as a highly efficient metal for various C-C and C-N cross-coupling reactions, as reported by Gosmini, Oshima, and others [32,33,34]. It has been found, in the literature, that the cobalt-catalyzed reactions were more specific compared to their Pd and Ni counterparts [32,33,34,35]. Moreover, it is evident in the literature that the cobalt-catalyzed cross-coupling reactions followed an alternative mechanism. For example, the oxidative coupling for the cobalt-catalyzed reaction occurred through a single-electron transfer [36,37], hence avoiding the side reactions that are commonly observed in the Pd and Ni-catalyzed reactions. Hence, high efficiency, cost effectiveness, and milder reaction conditions makes cobalt-catalyzed reactions a powerful tool for C-C and C-heteroatom bond formation. Additionally, our group focuses on exploring a simpler catalytic system for organic transformation [38]; in our continuing effort, a series of simpler ligands were also tested during the optimization of C-P bond formation in the current work. 

In the present work, we are pleased to disclose a simple, inexpensive, and efficient method for the synthesis of aryl phosphonates. The reaction has been performed in the presence of cobalt salt and zinc metal under mild reaction conditions in the presence of ter-pyridine as the ligand. To the best of our knowledge, this is the first cobalt-catalyzed C-P bond formation report so far.

## 2. Result and Discussion

Initially, we optimized the reaction by choosing a cobalt-based catalytic system for the cross-coupling reaction of phenylboronic acid with diethyl phosphite. The test reaction was carried out with CoCl_2_ (8 mol%) in the presence of Cs_2_CO_3_ (1 equiv) and ligand **1** (10 mol%) in acetonitrile at room temperature. No inert atmosphere was maintained while carrying out the reaction. The reaction proceeded with a poor yield (36%) and no further improvement was observed by increasing the reaction time up to 24 h. However, it was promising to see that the product formation was taking place. The reaction was also exposed to higher temperatures, ranging from 50 to 78 °C, but no increase in yield was observed. We then continued the screening of different bases such as K_2_CO_3_, Et_3_N, and DIPEA (entries 1–5, Table 1), but not much improvement was observed. However, when Zn powder (1 equiv) was added to the reaction, a drastic improvement in the yields was observed (entry 2 vs. entry 4, Table 1). We then continued to screen other cobalt-catalyst and ligands to optimize the reaction conditions further. The effect of different cobalt salts and ligands has been summarized in Table 1. It was found that the reaction proceeded with a good yield (84%, entry 12, Table 1) with cobalt (II) iodide (8 mol%) as the catalyst in the presence of zinc powder, and ligand **4**. When Co(NO_3_)_2_ was used as the catalyst (entry 11, Table 1), the reaction yield dropped to 61%. The use of Co(OAc)_2_ did not improve the reaction yield either (entry 13, Table 1) and only 56% of the cross-coupling product was obtained. When Co(acac)_2_ was used to carry out the reaction, 63% of the product was obtained.

However, when the reaction was carried out with cobalt (II) bromide (8 mol%), as the catalyst in the presence of zinc powder and ligand **4,** an excellent yield (91%) was obtained (entry 14, Table 1). It was also found that the reaction gave poor yields in the absence of zinc powder. We have also screened various ligands (entry 6–10, Table 1) and it was found that the presence of ter-pyridine (ligand **4**, entry 9, Table 1) effectively influenced the reaction outcome. The presence of a ligand increased the reaction yield significantly, as compared to the yield with no ligand. The reaction only worked moderately well when pyridine (ligand **1**), 2-picolylamine (ligand **2**), di-(2-picolyl)amine (ligand **3**), and 2,2′-dipyridylamine (ligand **5**) were used as ligands (as shown in Table 2), which clearly shows the crucial role of the ligand in this catalytic system. The reaction was also carried out with different catalyst loadings and it was found that the reaction worked best with 8 mol% of CoBr_2_. Further, a decrease in the catalyst loading adversely affected the reaction profile. For further optimizations, the effect of solvents on the reaction was observed, and after screening solvents such as THF, toluene, dimethylformamide, and 1,4-dioxane, acetonitrile, it was found that the reaction worked best with toluene as the solvent. 

With these optimized conditions in hand, the substrate scope for this reaction was tested by reacting a variety of arylboronic acids with diethyl phosphite. The reaction was carried out with boronic acid (0.20 mmol) reacting with diethyl phosphite (0.20 mmol) in the presence of 8 mol% of the cobalt-catalyst, ter-pyridine (10 mol%), and zinc powder (20 mol%). 

The effect of different groups on the arylboronic acid has been screened (Table 3) and it was found that both the presence of the electron-withdrawing group, as well as the electron-donating group, worked efficiently under the optimized reaction conditions. The presence of the trifluoromethyl group on the boronic acid decreased the reaction yield significantly (entry 9, Table 3). In the presence of ortho-substitutent, the reaction worked very well, with high yields in almost all cases (entries 2 and 7, Table 3). The reaction had good tolerance with a variety of functional group such as methoxy, nitro, and trifloromethyl (entries 3, 5, and 9, Table 3). 

After screening various boronic acids, a series of dialkyl/diaryl phosphites were also tested to see the scope of the reaction under the same optimized reaction conditions (Table 4). Additionally, we were pleased to find that the reaction worked very well with all the phosphite derivatives. The yields were high in alkyl and aryl phosphites. Diphenyl and dibenzyl phosphite gave good yields with the arylboronic acid, unlike copper-catalyzed reaction, where dibenzyl phosphonate ester resulted in moderate to poor yields [13].

**Scheme 1:**
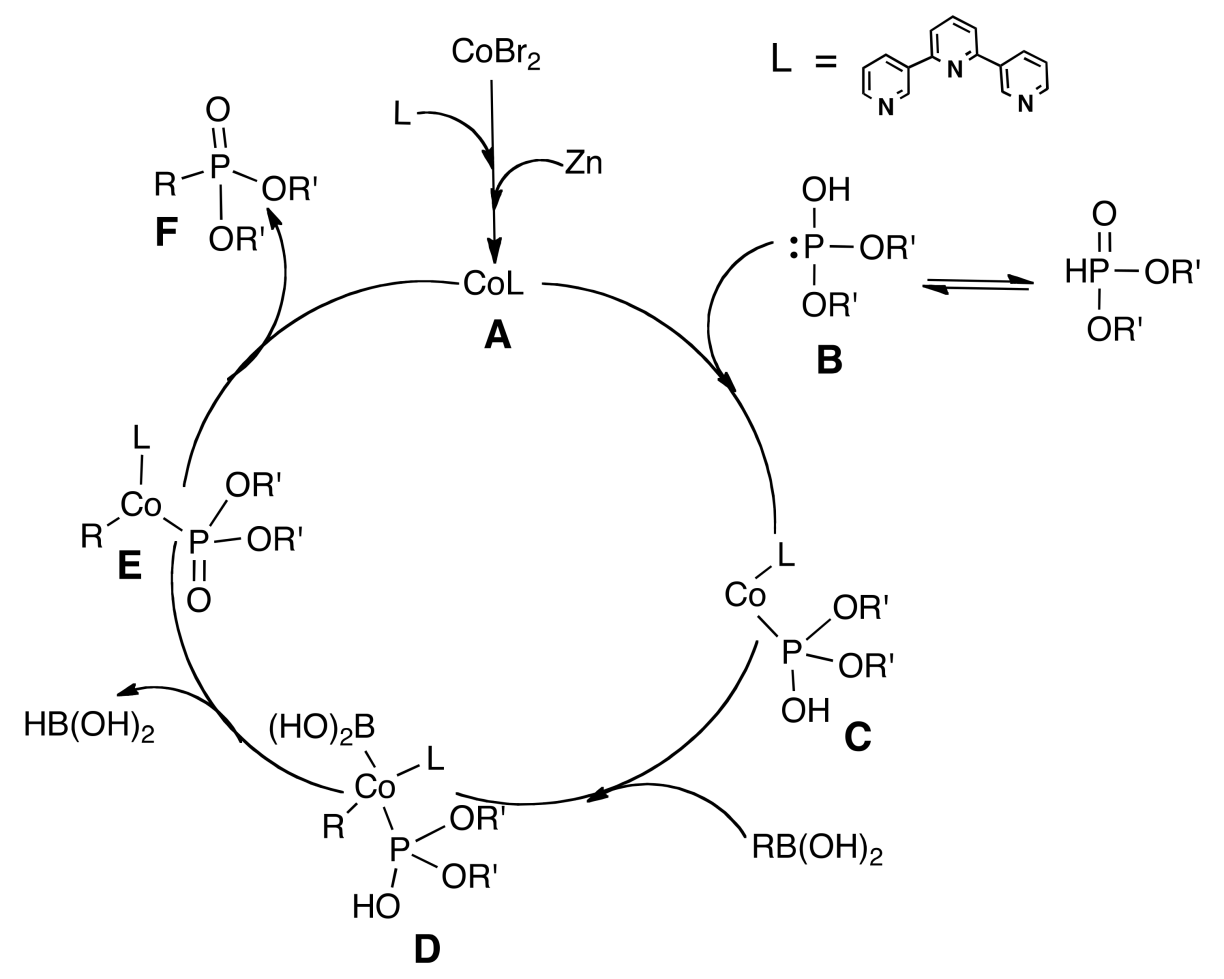
Proposed mechanism for the Co-Zn catalyzed cross-coupling.

A plausible mechanism has been proposed in Scheme 1. While an elaborated study is required to fully understand the mechanism of the reported methodology, it is anticipated [39] that the active catalyst was generated when cobalt (II) salt was generated in the presence of the zinc powder and ligand **4**. It is also anticipated that the active catalyst **A** then undergoes oxidative addition with the dialkyl phosphite (**B**) to generate the intermediate **C**, which, upon reaction with boronic acid, leads to the formation of intermediate **D**. The elimination of boronic acid from intermediate **D** then leads to the formation of intermediate **E**. Finally, the C-P bond forms by reductive elimination, which leads to the regeneration of active catalyst **A**. Alternatively, the addition of arylboronic acid to Co(0) in order to give Co(II) might be a possibility, which will be followed by transmetallation with the deprotonated phoshite, and finally, the reductive elimination leads to the formation of the cross-coupling product [31].

## 3. Materials and Methods

### 3.1. General Information

All experiments were carried out using dry solvents. The boronic acid, dialkyl phosphites, DIPEA, triethylamine, pyridine, 2,2′-dipyridylamine, 2-picolylamine, di-(2-picolyl)amine, and ter-pyridine, as well as the cobalt salts, were purchased from Sigma Aldrich. Silica gel for column chromatography and thin layer chromatography (TLC) plates (Silica gel 60 F_254_) were ordered from EMD/Merck. The analytical thin layer chromatography was eluted in the Ethylacetate/Hexane (30:70) solvent system. An ultra-violet lamp, iodine chamber, and PMA solution spray was used to visualize the product spots on thin layer chromatography (TLC). ^1^H-NMR and ^13^C-NMR spectra were recorded on Bruker Advance 400 and 100 MHz, respectively, and were referenced to residual protic solvent (CDCl3 ^1^H [7.26 ppm] and CDCl3 ^13^C [77.00 ppm]. Mass analyses were performed on the Joel GC-MS instrument (EI). The products were fully characterized using ^1^H-NMR, ^13^C-NMR, ^31^P-NMR, and GC-MS, and the relevant data has been provided in this section. All reagents were used as received from commercial sources without further purification. 

### 3.2. General Procedure for the Synthesis of Aryl Phosphonates

The phenyl boronic acid (**6a**, 0.20 mmol) was added to a 25 mL round bottom flask (oven-dried overnight), followed by the addition of the cobalt (II) bromide (8 mol%), ter-pyridine (ligand **4**, 10 mol%), and zinc powder (20 mol%). At this point the solvent, dry acetonitrile (6 mL), was added to the reaction mixture, followed by the addition of diethyl phosphite (**7b**, 0.20 mmol) which was added sequentially. The reaction vessel was covered with the septum and the reaction was stirred at room temperature. The reaction was allowed to run for 24 h at the same temperature. At this time, the reaction progress was monitored using TLC. The reaction was monitored at 4 h intervals and was ultimately left stirring at room temperature overnight. After completion, the reaction mixture was quenched and extracted with ethyl acetate and water. The organic layer was collected, and the aqueous layer was again extracted with ethyl acetate three more times. The combined organic layer was further washed with sodium bicarbonate and brine solution. The organic layer was then dried over sodium sulfate, and filtered and evaporated using rotavap. The crude compound obtained was further purified using column chromatography (EA:hexane 30:70). The pure product (**8a**) was obtained as thick oil with a 91% yield. The 1-H and 13-C data for the products obtained matched those reported previously [29,40,41].

#### 3.2.1. Compound **8a**: Diethyl Phenylphosphonate

^1^H-NMR (400 MHz, CDCl_3_): δ 7.54 (d, *J* = 7.0 Hz, 2H), 7.39 (t, *J* = 7.2 Hz, 2H), 7.32 (t, *J* = 7.0 Hz, 1H), 4.22 (m, 4H, CH2), 1.62 (td, *J_CH_*_3*-CH*2_ = 7.2 Hz, *J_CH_*_3*P*_ = 1.0 Hz, 6H). ^13^C-NMR (100 MHz, CDCl_3_): δ 131.6 (d, *J* = 3.0 Hz, C4), 130.9 (d, *J* = 8.0 Hz, 2C), 128.4 (d, *J* = 14.0 Hz, 2C), 125.1 (d, *J* = 178.4 Hz, C1), 64.3 (d, *J_CH_*_2_ = 7.8 Hz, 2C), and 18.1 (d, *J_CH_*_3_ = 6.4 Hz, 2C). GC-MS *m*/*z*: [M + H]^+^ Calcd for C_10_H_16_O_3_P 215.0832; Found 215.0815.

#### 3.2.2. Compound **8b**: Diethyl 2-Methylphenylphosphonate

^1^H-NMR (400 MHz, CDCl_3_): δ 7.44 (d, *J* = 8.0 Hz, 1H), 7.21 (d, *J* = 6.8 Hz, 1H), 7.09–7.02 (m, 2H) 3.84 (m, 4H, CH2), 1.32 (t, *J_CH_*_3*-CH*2_ = 7.4 Hz, 6H). ^13^C-NMR (100 MHz, CDCl_3_): δ 135.6 (d, *J* = 4.4 Hz, C4), 132.2 (d, *J* = 8.0 Hz, C3, C6), 128.4 (d, *J* = 3.2 Hz, C5), 126.4 (d, *J* = 4.8 Hz, C2), 123.1 (d, *J* = 183.6 Hz, C1), 65.8 (d, *J_CH_*_2_ = 6.8 Hz, 2C), 24.1 (d, *J_CH_*_3_ = 7.2 Hz, 2C), and 19.5. GC-MS *m*/*z*: [M + 1]^+^) calculated for C_11_H_18_O_3_P 228.0654; Found 229.0217.

#### 3.2.3. Compound **8c**: Diethyl 4-Methoxyphenylphosphonate

^1^H-NMR (400 MHz, CDCl_3_): δ 7.48 (d, *J* = 9.6 Hz, 2H), 7.22 (d, *J* = 10.0 Hz, 2H), 4.14 (m, 4H, CH2), 3.59 (s, 3H), 1.68 (t, *J_CH_*_3*-CH*2_ = 6.4 Hz, 6H). ^13^C-NMR (100 MHz, CDCl_3_): δ 165.2 (d, *J* = 4.6 Hz, C4), 133.4 (d, *J* = 2.8 Hz, C2, C6), 128.2 (d, *J* = 174.6 Hz, C1), 125.2 (d, *J* = 6.4 Hz, C3, C5), 70.1 (d, *J_CH_*_2_ = 6.2 Hz, 2C), and 53.2, 18.1 (d, *J_CH_*_3_ = 7.8 Hz, 2C). 

#### 3.2.4. Compound **8d**: Diethyl 4-Ethylphenylphosphonate

^1^H-NMR (400 MHz, CDCl_3_): δ 7.31 (d, *J* = 7.8 Hz, 2H), 7.17 (d, *J* = 8.0 Hz, 2H), 3.62 (m, 4H, CH2), 2.06 (m, 2H), 1.28 (t, *J_CH_*_3*-CH*2_ = 7.4 Hz, 6H), 1.22 (t, *J* = 6.8 Hz, 3H). ^13^C-NMR (100 MHz, CDCl_3_): δ 132.6 (d, *J* = 3.0 Hz, C4), 131.1 (d, *J* = 4.2 Hz, C2, C6), 127.1 (d, *J* = 4.0 Hz, C3, C5), 125.1 (d, *J* = 183.6 Hz, C1), 62.1 (d, *J_CH_*_2_ = 7.2 Hz, 2C), 22.1 (d, *J_CH_*_3_ = 6.8 Hz, 2C), 19.5, and 15.1. 

#### 3.2.5. Compound **8e**: Diethyl 3-Nitrophenylphosphonate

^1^H-NMR (400 MHz, CDCl_3_): δ 7.68 (s, 1H), 7.61 (d, *J* = 7.4 Hz, 1H), 7.54 (d, *J* = 5.6 Hz, 1H), 7.32 (t, *J* = 7.2 Hz, 1H), 3.78 (m, 4H, CH2), 1.29 (t, *J_CH_*_3*-CH*2_ = 8.0 Hz, 6H). ^13^C-NMR (100 MHz, CDCl_3_): δ 162.4 (d, *J* = 12.0 Hz, C5), 154.6 (d, *J* = 4.0 Hz, C4), 152.2 (d, *J* = 6.4 Hz, C6 ), 134.1 (d, *J* = 6.8 Hz, C2), 128.3 (d, *J* = 6.8 Hz, C3), 123.1 (d, *J* = 179.6 Hz, C1), 70.3 (d, *J_CH_*_2_ = 9.2 Hz, 2C), and 23.1 (d, *J_CH_*_3_ = 7.2 Hz, 2C). 

#### 3.2.6. Compound **8f**: Diethyl 4-Fluorophenylphosphonate

^1^H-NMR (400 MHz, CDCl_3_): δ 7.72 (d, *J* = 8.0 Hz, 2H), 7.47 (d, *J* = 7.6 Hz, 2H), 3.99 (m, 4H, CH2), 1.48 (t, *J_CH_*_3*-CH*2_ = 8.4 Hz, 6H). ^13^C-NMR (100 MHz, CDCl_3_): δ 142.4 (d, *J* = 176.0 Hz, C4), 138.6 (d, *J* = 3.0 Hz, C2, C6), 132.9 (d, *J* = 6.0 Hz, C3, C5), 123.1 (d, *J* = 183.6 Hz, C1), 68.3 (d, *J_CH_*_2_ = 8.4 Hz, 2C), and 20.1 (d, *J_CH_*_3_ = 7.2 Hz, 2C). GC-MS *m*/*z*: [M + 1]^+^) calculated for C_10_H_15_FO_3_P 232.0811; Found 232.0656.

#### 3.2.7. Compound **8h**: Diethyl 2,6-Difluorophenylphosphonate

^1^H-NMR (400 MHz, CDCl_3_): δ 7.32 (d, *J* = 6.4 Hz, 1H), 7.11 (d, *J* = 6.4 Hz, 1H), 7.02 (t, *J* = 7.8 Hz, 1H), 4.06 (m, 4H, CH2), 1.62 (t, *J_CH_*_3*-CH*2_ = 4.4 Hz, 6H). ^13^C-NMR (100 MHz, CDCl_3_): δ 176.6 (d, *J* = 4.4 Hz, C2, C6), 132.2 (d, *J* = 8.0 Hz, C3, C5), 126.1 (d, *J* = 174.2 Hz, C1), 125.6 (d, *J* = 6.0 Hz, C4), 61.8 (d, *J_CH_*_2_ = 5.4 Hz, 2C), and 22.6 (d, *J_CH_*_3_ = 6.8 Hz, 2C). 

#### 3.2.8. Compound **9a**: Dimethyl Phenylphosphonate

^1^H-NMR (400 MHz, CDCl_3_): δ 7.38 (d, *J* = 7.6 Hz, 2H), 7.31 (t, *J* = 7.0 Hz, 2H), 6.98 (t, *J* = 7.2 Hz, 1H), 3.84 (d, *J* = 11.0 Hz, 6H, CH3). ^13^C-NMR (100 MHz, CDCl_3_): δ 137.6 (d, *J* = 4.4 Hz, C4), 134.2 (d, *J* = 10.2 Hz, C2, C6), 129.2 (d, *J* = 12.6 Hz, C3, C5), 125.4 (d, *J* = 182.8 Hz, C1), and 55.8 (d, *J* = 6.0 Hz, 2C). GC-MS *m*/*z*: [M]^+^) calculated for C_8_H_11_O_3_P 186.0446, found 186.0432.

#### 3.2.9. Compound **9b**: Dimethyl 4-Methoxyphosphonate

^1^H-NMR (400 MHz, CDCl_3_): δ 7.22 (d, *J* = 7.2 Hz, 2H), 7.16 (t, *J* = 7.2 Hz, 2H), 6.77 (t, *J* = 6.8 Hz, 1H), 3.92 (d, *J* = 11.2 Hz, 6H, CH3), 3.45 (s, 3H). ^13^C-NMR (100 MHz, CDCl_3_): δ 132.2 (d, *J* = 4.0 Hz, C4), 130.6 (d, *J* = 10.6 Hz, C2, C6), 127.4 (d, *J* = 11.8 Hz, C3, C5), 125.2 (d, *J* = 188.2 Hz, C1), 55.8 (d, *J* = 6.0 Hz, 2C), and 52.4

#### 3.2.10. Compound **9c**: Dimethyl 3-Nitrophenylphosphonate

^1^H-NMR (400 MHz, CDCl_3_): δ 7.68 (s, 1H), 7.61 (d, *J* = 7.4 Hz, 1H), 7.54 (d, *J* = 5.6 Hz, 1H), 7.32 (t, *J* = 7.2 Hz, 1H), 3.62 (d, *J* = 11.2 Hz, 6H, CH3). ^13^C-NMR (100 MHz, CDCl_3_): δ 162.4 (d, *J* = 12.0 Hz, C5), 154.6 (d, *J* = 4.0 Hz, C4), 152.2 (d, *J* = 6.4 Hz, C6 ), 134.1 (d, *J* = 6.8 Hz, C2), 128.3 (d, *J* = 6.8 Hz, C3), 123.1 (d, *J* = 179.6 Hz, C1), 70.3 (d, *J_CH_*_2_ = 9.2 Hz, 2C), and 23.1 (d, *J_CH_*_3_ = 7.2 Hz, 2C). 

#### 3.2.11. Compound **10a**: Diisopropyl Phenylphosphonate

^1^H-NMR (400 MHz, CDCl_3_): δ 7.58 (d, *J* = 7.8 Hz, 3H), 7.31 (t, *J* = 7.2 Hz, 2H), 4.69–4.51 (m, 2H), 1.31 (d, *J* = 6.3 Hz, 6H), 1.12 (d, *J* = 6.3 Hz, 6H).^13^C-NMR (100 MHz, CDCl_3_): δ 142.4 (d, *J* = 12.8 Hz, C5), 138.4 (d, *J* = 5.2 Hz, C2, C4), 135.6 (d, *J* = 6.8 Hz, C6 ), 128.3 (d, *J* = 6.8 Hz, C3), 123.1 (d, *J* = 181.6 Hz, C1), 69.8 (d, *J_CH_*_2_ = 9.2 Hz, 2C), and 24.2 (d, *J_CH_*_3_ = 7.2 Hz, 2C). 

#### 3.2.12. Compound **10b**: Diisopropyl 4-Methoxyphosphonate

^1^H-NMR (400 MHz, CDCl_3_): δ 7.51 (d, *J* = 7.4 Hz, 2H), 7.44 (d, *J* = 7.6 Hz, 2H), 4.48–4.36 (m, 2H), 3.90 (s, 3H), 1.37 (d, *J* = 7.2 Hz, 6H), 1.21 (d, *J* = 7.8 Hz, 6H). ^13^C-NMR (100 MHz, CDCl_3_): δ 156.4 (d, *J* = 6.8 Hz, C4), 143.8 (d, *J* = 6.4 Hz, C3, C5 ), 132.3 (d, *J* = 6.8 Hz, C2, C6), 123.1 (d, *J* = 179.6 Hz, C1), 71.4 (d, *J_CH_*_2_ = 10.2 Hz, 2C), 52.8 (d, *J* = 8.2 Hz, OCH_3_), and 24.2 (d, *J_CH_*_3_ = 7.2 Hz, 2C). 

#### 3.2.13. Compound **10c**: Diisopropyl 3-Nitrophenylphosphonate

^1^H-NMR (400 MHz, CDCl_3_): δ 7.39 (s, 1H), 7.61 (d, *J* = 7.4 Hz, 1H), 7.54 (d, *J* = 5.6 Hz, 1H), 7.22 (t, *J* = 7.0 Hz, 1H), 4.59–4.66 (m, 2H), 1.34 (d, *J* = 6.3 Hz, 6H), 1.19 (d, *J* = 6.3 Hz, 6H). ^13^C-NMR (100 MHz, CDCl_3_): δ 161.3 (d, *J* = 11.0 Hz, C5), 158.6 (d, *J* = 4.4 Hz, C4), 151.0 (d, *J* = 6.8 Hz, C6), 132.4 (d, *J* = 7.2 Hz, C2), 129.1 (d, *J* = 6.2 Hz, C3), 125.4 (d, *J* = 178.2 Hz, C1), 70.5 (d, *J_CH_*_2_ = 10.0 Hz, 2C), and 23.5 (d, *J_CH_*_3_ = 7.2 Hz, 2C). 

#### 3.2.14. Compound **11a**: Diphenyl Phenylphosphonate

^1^H-NMR (400 MHz, CDCl_3_): δ 7.76 (m, 3H), 7.54 (m, 2H), 7.31–7.23 (m, 4H), 7.11–7.06 (m, 6H). ^13^C-NMR (100 MHz, CDCl_3_): δ 160.8 (d, *J* = 12.0 Hz), 156.4 (d, *J* = 4.4 Hz), 150.6 (d, *J* = 6.4 Hz), 142.6 (d, *J* = 8.0 Hz), 141.6 (d, *J* = 6.6 Hz), 138.2 (d, *J* = 7.0 Hz), 135.7 (d, *J* = 8.4 Hz), 132.7 (d, *J* = 8.0 Hz), 128.3 (d, *J* = 6.2 Hz,), and 123.1 (d, *J* = 189.6 Hz). 

#### 3.2.15. Compound **11b**: Diphenyl 4-Methoxyphosphonate

^1^H-NMR (400 MHz, CDCl_3_): δ 7.56 (dd, *J*_1_ = 13.2 Hz, *J*_2_ = 8.7 Hz, 2H), 7.21–7.19 (m, 5H), 7.17–7.08 (m, 5H), 6.93 (m, 2H), 3.65 (s, 3H). ^13^C-NMR (100 MHz, CDCl_3_): δ 159.4 (d, *J* = 12.2 Hz), 155.2 (d, *J* = 4.8 Hz), 150.4 (d, *J* = 7.4 Hz), 142.3 (d, *J* = 7.4 Hz), 138.6 (d, *J* = 8.4 Hz), 132.5 (d, *J* = 8.6 Hz), 129.4 (d, *J* = 7.4 Hz), 127.2 (d, *J* = 7.0 Hz), 124.6 (d, *J* = 174.6 Hz), 122.1 (d, *J* = 8.4 Hz), and 54.3 (d, *J_CH_*_2_ = 10.4 Hz, OCH_3_). 

#### 3.2.16. Compound **11c**: Diphenyl 3-Nitrophenylphosphonate

^1^H-NMR (300 MHz, CDCl_3_) δ 7.83 (m, 4H), 7.21–7.19 (m, 4H), 7.17–7.08 (m, 3H), 6.93 (m, 3H). ^13^C-NMR (100 MHz, CDCl_3_): δ 166.5 (d, *J* = 12.2 Hz), 156.8 (d, *J* = 4.8 Hz), 151.8 (d, *J* = 6.4 Hz), 132.1 (d, *J* = 7.2 Hz), 130.4 (d, *J* = 7.0 Hz), 128.7 (d, *J* = 6.2 Hz), 127.9 (d, *J* = 6.8 Hz), 125.9 (d, *J* = 7.1 Hz), 123.9 (d, *J* = 186.6 Hz), 122.6 (d, *J* = 7.4 Hz), and 119.6 (d, *J* = 7.2 Hz). 

#### 3.2.17. Compound **12a**: Dibenzyl Phenylphosphonate

1H-NMR (400 MHz, CDCl_3_): δ 7.72 (m, 4H), 7.21–7.14 (m, 9H), 6.98 (dd, *J1* = 9.2 Hz, *J2* = 3.4 Hz, 2H), 5.09–4.91 (m, 4H); 13C-NMR (100 MHz, CDCl_3_): δ 161.0 (d, *J* = 3.2 Hz), 155.6 (d, *J* = 4.6 Hz), 153.4 (d, *J* = 4.2 Hz), 145.3 (d, *J* = 8.0 Hz), 137.4 (d, *J* = 8.0 Hz), 133.7 (d, *J* = 10.0 Hz), 128.5 (d, *J* = 8.4 Hz), 128.2 (d, *J* = 7.8 Hz), 127.9 (d, *J* = 6.4 Hz), 119.0 (d, *J* = 189.8 Hz), 114.1 (d, *J1* = 14.0 Hz), and 67.4 (d, *J* = 50.0 Hz). 

#### 3.2.18. Compound **12b**: Dibenzyl 4-Methoxyphosphonate

1H-NMR (400 MHz, CDCl_3_): δ 7.65 (d, *J* = 12.4 Hz, 3H), 7.18–7.08 (m, 8H), 6.93 (dd, *J1* = 9.4 Hz, *J2* = 4.0 Hz, 3H), 4.76–4.51 (m, 4H); 13C-NMR (100 MHz, CDCl_3_): δ 163.0 (d, *J* = 4.0), 154.6 (d, *J* = 7.2 Hz), 152.8 (d, *J* = 7.2 Hz), 147.6 (d, *J* = 7.2 Hz), 140.4 (d, *J* = 7.2 Hz), 138.2 (d, *J* = 7.6 Hz), 133.5 (d, *J* = 8.0 Hz), 134.3 (d, *J* = 10.0 Hz), 130.2 (d, *J* = 6.2 Hz), 129.1 (d, *J* = 5.6 Hz), 124.9 (d, *J* = 8.0 Hz), 121.0 (d, *J* = 181.4 Hz), 113.1 (d, *J* = 12.8 Hz), and 59.4 (d, *J* = 22.0 Hz), 58.4.

#### 3.2.19. Compound **12c**: Dibenzyl 3-Nitrophenylphosphonate

1H-NMR (400 MHz, CDCl_3_): δ 7.55 (m, 3H), 7.20–7.17 (m, 7H), 7.03 (m, 2H), 6.78 (dd, *J1* = 8.0 Hz, *J2* = 4.2 Hz, 2H), 5.11–5.01 (m, 4H); 13C-NMR (100 MHz, CDCl_3_):δ 161.0 (d, *J* = 3.8 Hz), 152.6 (d, *J* = 7.0 Hz), 151.0 (d, *J* = 7.8 Hz), 145.6 (d, *J* = 5.6 Hz), 142.2 (d, *J* = 8.0 Hz), 135.1 (d, *J* = 8.2 Hz), 132.1 (d, *J* = 8.2 Hz), 130.1 (d, *J* = 6.8 Hz), 129.7 (d, *J* = 6.2 Hz), 123.4 (d, *J* = 6.8 Hz), 122.4 (d, *J* = 180.8 Hz), 112.1 (d, *J* = 11.0 Hz), and 58.4 (d, *J* = 7.6 Hz). 

#### 3.2.20. Compound **12d**: Dibenzyl 4-(Trifluoromethyl)phenylphosphonate

1H-NMR (400 MHz, CDCl_3_): δ 7.48 (m, 3H), 7.16–7.07 (m, 8H), 6.73 (dd, *J1* = 10.0 Hz, *J2* = 5.2 Hz, 3H), 4.98–4.75 (m, 4H); 13C-NMR (100 MHz, CDCl_3_): δ 168.2 (d, *J* = 5.2 Hz), 155.2 (d, *J* = 5.6 Hz), 153.3 (d, *J* = 8.4 Hz), 149.7 (d, *J* = 6.0 Hz), 144.3 (d, *J* = 7.4 Hz), 139.0 (d, *J* = 8.6 Hz), 135.1 (d, *J* = 8.8 Hz), 132.4 (d, *J* = 7.0 Hz), 129.4 (d, *J* = 5.8 Hz), 125.2 (d, *J* = 7.8 Hz), 123.4 (d, *J* = 183.5 Hz), 115.1 (d, *J* = 11.2 Hz), and 59.4 (d, *J* = 7.8 Hz).

## 4. Conclusions

In conclusion, we have reported the first cobalt-catalyzed cross-coupling of arylboronic acid with alkyl/aryl phosphites under mild conditions in the presence of zinc powder. Moderate to high yields of the products were observed in almost all the cases screened. This new protocol is advantageous because it uses a non air-sensitive ligand, and is an inexpensive and non-toxic cobalt-catalyst. The ligands involved are much simpler and less bulky than the ones that are employed in Pd, Cu, or –Ni catalyzed reactions; hence, the proposed method provides a better alternative for such transformations. The reaction also has a great substrate scope with arylboronic acid, as well as with different dialkyl/diaryl phosphites, which broadens its scope and can make it a widely used method. Further studies regarding the exact mechanistic pathway and future scope of this catalyst system are still under way.

## Figures and Tables

**Table 1:**
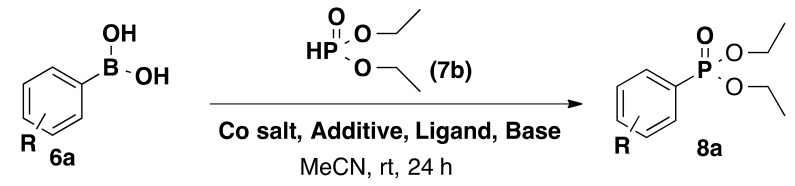
Optimization of the reaction conditions ^a^.

Entry	Ligand	Cobalt Source	Additive	Base	Yield%
1	1	CoCl_2_	-	Cs_2_CO_3_	19
2	1	CoCl_2_	-	K_2_CO_3_	32
3	1	CoCl_2_	Zn	DIPEA	34
4	1	CoCl_2_	Zn	K_2_CO_3_	38
5	1	CoCl_2_	Zn	Et_3_N	44
6	1	CoCl_2_	Zn	-	42
7	2	CoCl_2_	Zn	-	56
8	3	CoCl_2_	Zn	-	65
9	4	CoCl_2_	Zn	-	79
10	5	CoCl_2_	Zn	-	60
11	4	Co(NO_3_)_2_	Zn	-	61
12	4	CoI_2_	Zn	-	84
13	4	Co(OAc)_2_	Zn	-	56
14 15	4 4	CoBr_2_ Co(acac)_2_	Zn Zn	- -	91 63

^a^ All reactions were carried out at room temperature with Boronic acid (0.20 mmol), Co salt (8 mol%), ligand (10 mol%), additive (20 mol%), and dialkylphosphite (0.20 mmol) in dry acetonitrile for 24 h.

**Table 2:** Screening of various ligands for the C-P bond formation reaction ^a^.

Entry	Ligand	% Yield
1	Pyridine (ligand **1**)	54
2	2-picolylamine (ligand **2**)	56
3	di-(2-picolyl)amine (ligand **3**)	65
4	ter-pyridine (ligand **4**)	79
5	2,2′-dipyridylamine (ligand **5**)	60

^a^ All reactions were carried out at room temperature with Boronic acid (0.20 mmol), Co salt (8 mol%), ligand (10 mol%), additive (20 mol%), and dialkylphosphite (0.20 mmol) in dry acetonitrile for 24 h.

**Table 3:**
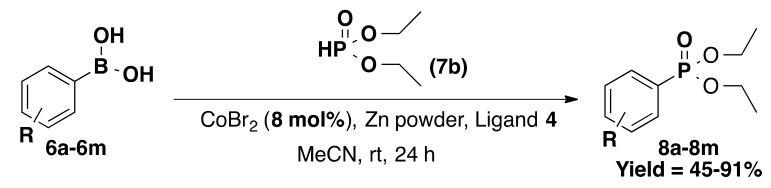
Substrate scope for the cross-coupling reaction between boronic acids (**6a–6j**) and diethyl phosphite (**7b**) ^a^.

Entry	R	Product	Yield%
1	H (**6a**)	**8a**	91
2	2-methyl (**6b**)	**8b**	89
3	4-methoxy (**6c**)	**8c**	87
4	4-ethynyl (**6d**)	**8d**	78
5	3-nitro (**6e**)	**8e**	66
6	4-fluoro (**6f**)	**8f**	65
7	2-fluoro (**6g**)	**8g**	82
8	2,6-difluoro (**6h**)	**8h**	63
9	4-trifluoromethyl (**6i**)	**8i**	45
10 11 12 13	1-Naphthyl (**6j**) 4-nitro (**6k**) 4-cyano (**6l**) 4-pyridyl (**6m**)	**8j** **8k** **8l** **8m**	76 74 68 57

^a^ All reactions were carried out at room temperature with aldehyde (0.20 mmol), Co salt (8 mol%), ligand (10 mol%), Zn powder (20 mol%), and dialkylphosphite (0.20 mmol) in dry acetonitrile for 24 h.

**Table 4:**
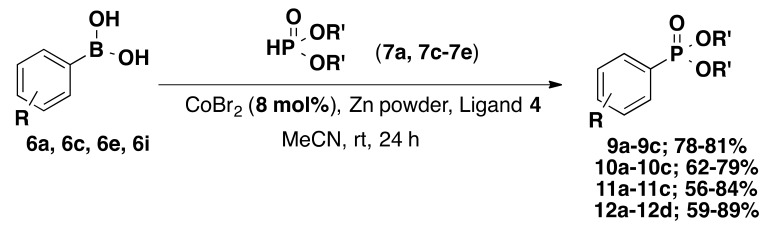
Results of screening different dialkyl/diaryl phosphites ^a^.

Entry	R	R′	Product	Yield%
1	H (**6a**)	Me (**7a**)	**9a**	79
2	4-methoxy (**6c**)	Me (**7a**)	**9b**	78
3	3-nitro (**6e**)	Me (**7a**)	**9c**	81
4	H (**6a**)	*i*Pr (**7c**)	**10a**	79
5	4-methoxy (**6c**)	*i*Pr (**7c**)	**10b**	62
6	3-nitro (**6e**)	*i*Pr (**7c**)	**10c**	71
7	H (**6a**)	Ph (**7d**)	**11a**	84
8	4-methoxy (**6c**)	Ph (**7d**)	**11b**	74
9	3-nitro (**6e**)	Ph (**7d**)	**11c**	56
10	H (**6a**)	Bn (**7e**)	**12a**	89
11	4-methoxy (**6c**)	Bn (**7e**)	**12b**	78
12	3-nitro (**6e**)	Bn (**7e**)	**12c**	67
13	4-trifluoro(methyl) (**6i**)	Bn (**7e**)	**12d**	59

^a^ All reactions were carried out at room temperature with aldehyde (0.20 mmol), Co salt (8 mol%), ligand (10 mol%), Zn powder (20 mol%), and dialkylphosphite (0.20 mmol) in dry acetonitrile for 24 h.
